# NETopathies? Unraveling the Dark Side of Old Diseases through Neutrophils

**DOI:** 10.3389/fimmu.2016.00678

**Published:** 2017-01-11

**Authors:** Alexandros Mitsios, Athanasios Arampatzioglou, Stella Arelaki, Ioannis Mitroulis, Konstantinos Ritis

**Affiliations:** ^1^Laboratory of Molecular Hematology, Democritus University of Thrace, Alexandroupolis, Greece; ^2^Department of Pathology, University General Hospital of Alexandroupolis, Alexandroupolis, Greece; ^3^Department of Clinical Pathobiochemistry, Institute for Clinical Chemistry and Laboratory Medicine, Faculty of Medicine Technische Universität Dresden, Dresden, Germany; ^4^First Department of Internal Medicine, University Hospital of Alexandroupolis, Democritus University of Thrace, Alexandroupolis, Greece

**Keywords:** neutrophil extracellular traps, neutrophil, thromboinflammation, autoimmunity, autoinflammation

## Abstract

Neutrophil extracellular traps (NETs) were initially described as an antimicrobial mechanism of neutrophils. Over the last decade, several lines of evidence support the involvement of NETs in a plethora of pathological conditions. Clinical and experimental data indicate that NET release constitutes a shared mechanism, which is involved in a different degree in various manifestations of non-infectious diseases. Even though the backbone of NETs is similar, there are differences in their protein load in different diseases, which represent alterations in neutrophil protein expression in distinct disorder-specific microenvironments. The characterization of NET protein load in different NET-driven disorders could be of significant diagnostic and/or therapeutic value. Additionally, it will provide further evidence for the role of NETs in disease pathogenesis, and it will enable the characterization of disorders in which neutrophils and NET-dependent inflammation are of critical importance.

## Introduction

Neutrophils constitute an essential part of the innate immune system in host defense against pathogens, as shown more than 100 years ago (1, 2). Circulating neutrophils are recruited in vast numbers at the sites of infection or sterile inflammation, in response to a variety of pathogen and host-derived inflammatory mediators (3). There, *via* adhesive interactions with endothelial cells, neutrophils rapidly infiltrate the site of inflammation (4). Uncontrolled inflammation in turn results in the release of newly produced neutrophils from the bone marrow, in a process termed as emergency granulopoiesis (5).

In addition to phagocytosis and degranulation, it has been recently proposed that neutrophils employ an additional strategy, in order to restrain infection: the release of NETs (1, 2, 6, 7).

Neutrophil extracellular traps are extracellular chromatin structures, formed upon certain inflammatory stimuli and composed of cytoplasmic, granular, and nuclear components of neutrophils (1, 2, 6, 7). To date, it is known that they can entrap and possibly kill pathogens. It has been shown that NETs bind bacteria (6, 8, 9) as well as fungi (10). The antimicrobial activity of NETs relies on both cytoplasmic and granular proteins as well as histones. This suggests that the intact NET structure is crucial for their antimicrobial function, enabling the increased local concentration of antimicrobial factors (1, 2, 6, 7, 11).

Besides their role in infectious disorders, studies carried out after 2008 support the role of NETs in the pathophysiology of non-infectious diseases, such as thrombosis (12–16), autoimmune diseases (14, 17–22), genetically driven autoinflammatory (23), and other inflammation-related diseases (24–26), metabolic disorders (27, 28), lung diseases (29–32), fibrosis (33), and cancer (34–36).

Herein, we seek to review current data regarding the proposed role of NETs in non-infectious human diseases. We also discuss the existing evidence supporting that these structures constitute a common mechanism of the pathophysiology of distinct diseases.

## Mechanism of NET Formation

Despite the morphological similarities of NETs released by neutrophils in response to different stimuli and under diverse conditions, it is nowadays widely accepted that there is more than one mechanism involved in NET release (37). Additionally, mitochondrial DNA also contributes in NET formation (38, 39), whereas, even though *in vitro* NET formation leads to cell death (40), it is reported that neutrophils that undergo NET release *in vivo* may remain active and functional, suggesting that NET formation may not necessarily be a terminal event (41, 42).

**Figure 1:**
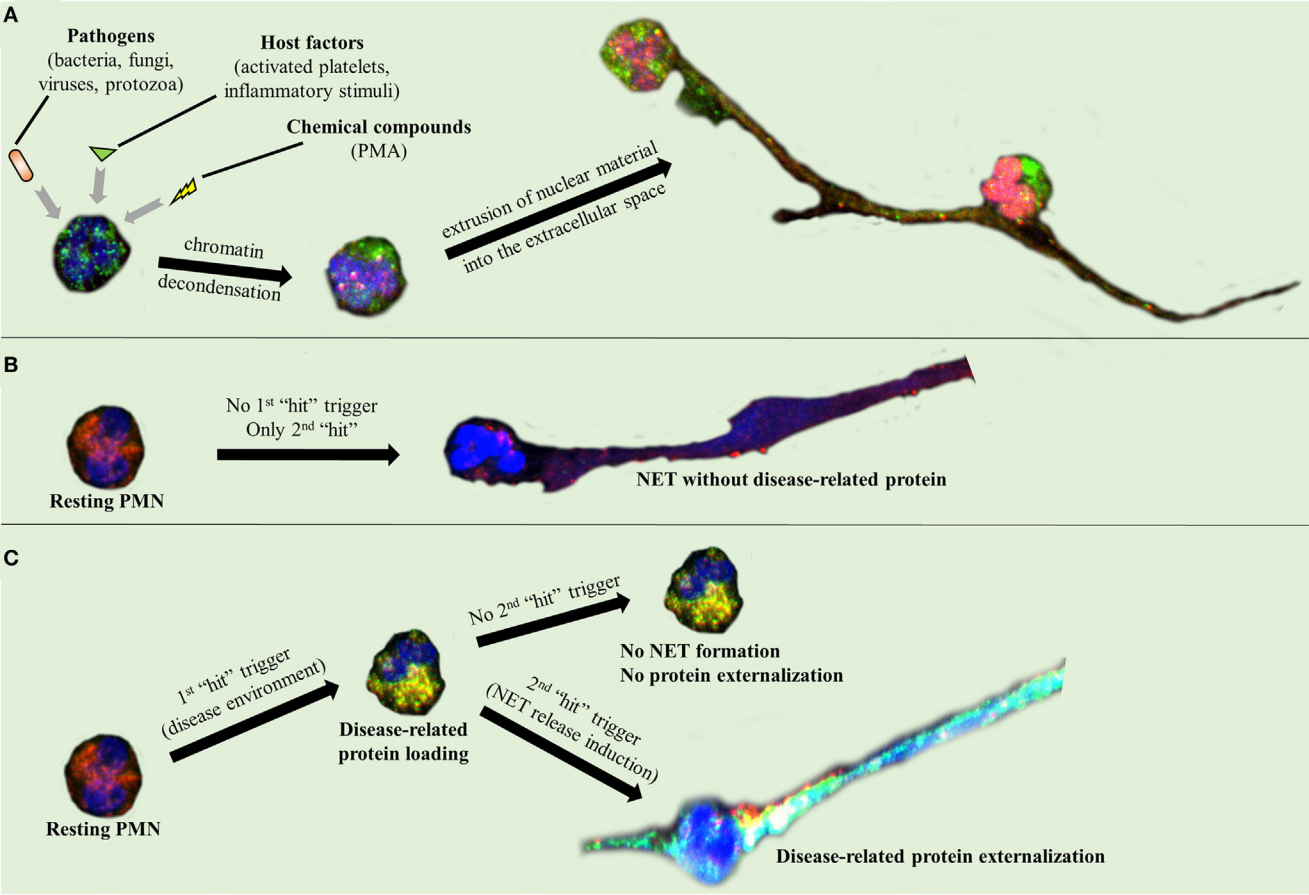
**Neutrophil extracellular trap (NET) formation and protein decoration**. Representative images taken using confocal microscopy, demonstrating **(A)** NET formation mechanism and **(B,C)** the two-step process through which the disease-related protein is externalized.

Activated neutrophils undergo dramatic morphological changes in order to release NETs (43–47). The nuclear and granular membranes disintegrate and elastase enters into the nucleus, followed by hypercitrullination of histones, chromatin decondensation into the cytoplasm, rupture of the plasma membrane, and extrusion of nuclear material from the cell into the extracellular space (43–47). The enzymes peptidyl arginine deiminase type IV (PAD4), neutrophil elastase (NE), and myeloperoxidase (MPO) have been implicated in the initial chromatin decondensation and in the degradation of the nuclear envelope (43–47). As a final step, extracellular DNA, histones, and granular enzymes form a network of NETs that entrap endogenous (e.g., platelets) and extrinsic (e.g., bacteria) particles and molecules (Figure 1A) (43–47). The negatively charged DNA acts as the backbone of the NET, interacting with other NET components through positive electrostatic charge (43–47). As it has been recently described, this scaffold is crucial for NET proteins to maintain their function (6, 43, 47), since dismantling of NET structures by DNase abolishes their antimicrobial activity (6). However, in the majority of these studies, PMA was used as a NET inducer (48, 49). Based on the criticism directed against the use of PMA as a NET inducer, the exact intracellular pathway that leads to NET release is still unclear (50).

At the molecular level, NET formation is still poorly understood and it is not defined whether neutrophils employ a similar mechanism to release NETs under different circumstances. However, there is strong evidence that the production of reactive oxygen species (ROS), the relocation of NE and MPO into the nucleus, histone citrullination, and eventually the rupture of the plasma membrane are, sequentially, involved in NETosis (43–47).

Cell metabolism has major contribution in immune cell function (51), including neutrophil activation. Neutrophils rely mainly on glycolysis for their metabolic needs, enabling their adaptation to the highly hypoxic inflammatory sites (52, 53).

Moreover, there is evidence that the metabolic shift to the pentose phosphate pathway is important for NET release, due to the involvement of glucose-6-phosphate dehydrogenase in fueling NADPH oxidase-2 with NADPH, to produce an effective amount of ROS and thus induce NETs. In contrast, mitochondrial ROS release, which is NADPH-independent, is not effective in signaling for NET production (54, 55).

Additionally, NET formation has been shown to require, at least in certain circumstances, the activation of autophagy (56). Autophagy is an anti-apoptotic mechanism activated in response to cell stress, in order to regulate protein and organelle turnover, ensuring cell survival (57). The protein kinase mammalian target of rapamycin (mTOR) negatively regulates autophagy, involved also in NET formation (58, 59). We and others (23, 24, 33, 56, 60–62) have shown that blocking autophagy through PI3K signaling, either at the initial levels by using 3-methyladenine (24, 60–62) or at the level of autophagosomal acidification by using wortmannin or bafilomycin (23, 33, 56, 62), inhibits the induction of NET release. However, more mechanistic studies are needed to identify how autophagy is involved in NET release, even though mTOR signaling and ROS production have been linked to both processes (7, 56, 59).

It is suggested that autophagy is crucial for NET release in both infectious and non-infectious diseases, including sepsis, familiar Mediterranean fever (FMF), gout, and inflammatory-driven fibrosis (12, 23, 56).

## Can NET Cargo Define Neutrophil Role in Disease?

Independently of the stimulus, NETs are composed of DNA, citrullinated histone 3 (cit-H3), NE, and MPO, the three main proteins commonly used for their detection (43–45, 47). Even though a proteomic analysis of infiltrating neutrophils in diverse tissues and in different disorders could be the proof of concept, there is evidence proposing that neutrophils express and release in the form of NETs a variety of proteins, depending on the specific inflammatory environment (63). For example, tissue factor (TF) was detected on NETs in vein and arterial thrombosis (16, 64, 65), interleukin 1 beta (IL-1β) in gouty arthritis (24) and FMF (23), interleukin 17 (IL-17) in psoriasis (66) and pulmonary fibrosis (33), antimicrobial peptide LL-37 in systemic lupus erythematosus (SLE) (19), and PAD4 in rheumatoid arthritis (RA) (67).

Even though NETs constitute a common event in distinct pathophysiologic conditions, the expression of distinct bioactive proteins on NETs in different disorders might be the one that determines their specific function in disease pathogenesis.

A two-“hit” process has been proposed to explain the differential protein cargo of NETs in distinct disorders. The first “hit” in this process is the disease-specific environment that primes neutrophils to express disease-associated protein. A second “hit” is then required for the induction of NET formation (Figures 1B,C). However, this is a simplified model, and we cannot exclude the possibility that the same stimulus can drive both events. A typical paradigm of this two-“hit” model has been described in ST-segment elevation acute myocardial infarction (16). It has been shown in acute coronary syndromes that a variety of inflammatory stimuli trigger the cytoplasmic expression of TF in circulating neutrophils. At sites of atherosclerotic plaque rupture, locally activated platelets interact with TF-loaded neutrophils leading to the release of TF-bearing NETs inside the affected artery. The release of functional TF on NETs is able to further induce thrombin generation and platelet activation, creating a possible vicious cycle, that leads to thrombus propagation and stability (16).

The expression of these “disease-related” proteins on NETs could increase their local bioactivity (12, 14, 16, 23, 66, 68). On the other hand, it has been shown that, at high densities, NETs limit inflammation by degrading cytokines and chemokines (69). This balance between the pro-inflammatory and prothrombotic role of NETs, though the expression of cytokines like IL-1β and IL-17, and their anti-inflammatory role, could be exploited for the development of new therapeutic approaches.

In the following section, we review the clinical and experimental data that link NETs with pathogenesis of several disorders. Even though the list of diseases in which NETs have been identified is extensive, we believe that the further characterization of the degree of NET involvement in such disorders could enable the classification of diseases in which NETs have a definite and strong involvement under the term of “NET-driven disorders” or “NETopathies.” The term NETopathy(ies) is derived from the abbreviation NET and the Greek word πάθος = *pathos*, which means disorder.

### NETs in Thromboinflammation

The widely accepted cross talk between inflammation and thrombosis has led to the introduction of the term thromboinflammation (68). Cells of the hematopoietic system, including neutrophils, platelets, and monocytes, have a major role in this process (64). There is increasing evidence implicating NET release with the development of both vein and arterial thrombosis (12, 14, 16, 26, 65, 70–77). Extracellular deposition of DNA co-localized with neutrophil granule proteins has been shown in thrombi from patients with deep vein thrombosis (DVT) (78), especially at the phase of organization of the thrombus (70). Additionally, circulating extracellular DNA in the form of nucleosomes and DNA associated with neutrophil granule proteins, supporting the induction of NET release, has been identified in blood samples from patients with DVT (79, 80). Similarly, NETs have been identified in thrombus specimens from patients undergoing thrombectomy in the context of myocardial infarction (15, 16, 62, 71). In a recent multicenter study in patients presenting with stent thrombosis, neutrophils were the more abundant leukocyte population in thrombus specimens, whereas NETs were identified in 23% of thrombi (71). Regarding specific disorders associated with thrombotic manifestations, NETs in thrombus specimens and/or increased levels of nucleosomes have been identified in disseminated intravascular coagulation in sepsis (73), in paroxysmal nocturnal hemoglobinuria (81), thrombotic microangiopathies (82), antiphospholipid syndrome (APS) (74), antineutrophil cytoplasmic antibody (ANCA)-associated vasculitis (AAV) (14), or hemodialysis-related thrombogenicity (83). These clinical data support a role for NETs in the development of both arterial and venous thrombosis.

The prothrombotic role of NETs was further confirmed in several experimental animal models. NETs were observed in thrombi, in a baboon model (75) and in several mouse models of DVT (64, 76, 84). In a mouse model of DVT, infusion of DNase I resulted in protection from thrombosis (76), whereas PAD4^−/−^ mice were protected from thrombosis (85), supporting the pathogenetic role of NETs in venous thrombosis, at least in this animal model. The *in vivo* role of NETs in the development of thrombosis was further shown in a mouse model of APS (86). Additionally, NETs contribute in cancer-induced venous thrombosis, as shown in a mouse model of chronic myelogenous leukemia (34) and in the RIP1-Tag2 model of insulinoma and MMTV-PyMT model of breast cancer (77). Brill et al. linked histones with the prothrombotic effect of NETs, since histone infusion also resulted in thrombosis. However, there is evidence that NETs participate in DVT *via* interaction with von Willebrand factor, a factor that potentially activates platelets (76). Furthermore, it has been reported that in a mouse model of DVT TF triggers intraluminal fibrin formation, while the release of NETs activates factor XII, consolidating DVT (64). The involvement of NET-bound TF, which is the main *in vivo* initiator of coagulation (87), in NET-dependent thromboinflammation has been shown in several studies, since TF has been identified in NETs released in neutrophils from patients with sepsis, APS, AAV, or myocardial infarction (12, 14, 16, 74) or in a mouse model of DVT (64).

The interplay between neutrophils and platelets has been shown to have a major contribution in NET release (16, 62, 72, 84, 88). Clark et al. have shown that upon toll-like receptor 4 (TLR4) activation platelets induce the formation of NETs in a mouse model of sepsis (72). This leads not only to bacterial but also to platelet entrapment in NETs, resulting in tissue damage (72). Several studies have further identified platelet derived high mobility group box 1 (HMGB1) as the factor that mediates platelet–neutrophil interaction and NET release (62, 84). HMGB1 released by platelets has been shown to promote thrombosis in a mouse model of DVT (84), whereas it mediates neutrophil activation in the context of myocardial infarction (62). The importance of platelet–neutrophil interaction is prominent in coronary artery thrombosis, since it was proved that coronary thrombi are mainly composed of interacting neutrophils and platelets (16, 62). The rupture of the atherosclerotic plaque primes a cascade of events, which results in platelet activation and NET release, leading to thrombus formation and blood vessel occlusion. The expression of TF on NETs may propagate the further activation of the coagulation system, leading to thrombus expansion (16).

Taken together, there is strong evidence, derived by clinical and experimental observation, that neutrophils and NETs are major players in both venous and arterial thrombosis. The development and clinical use of factors that target NETs could provide, however, the definite proof for the role of NETs in thrombotic disorders.

### NETs in Autoimmune Diseases

A growing number of studies demonstrate that NETs play a driving role in the pathogenesis of a variety of autoimmune disorders, such as SLE, AAV, RA, and psoriasis. In the aforementioned disorders, NETs are a main source of autoantigens, are present in excess amount, or are decorated with disease-specific proteins.

#### Systemic Lupus Erythematosus

Systemic lupus erythematosus is a systemic autoimmune disease and a well-studied model. SLE is characterized by systemic production of autoantibodies against a plethora of intracellular and extracellular targets. These autoantibodies are able to cause extensive tissue damage (89, 90).

**Figure 2:**
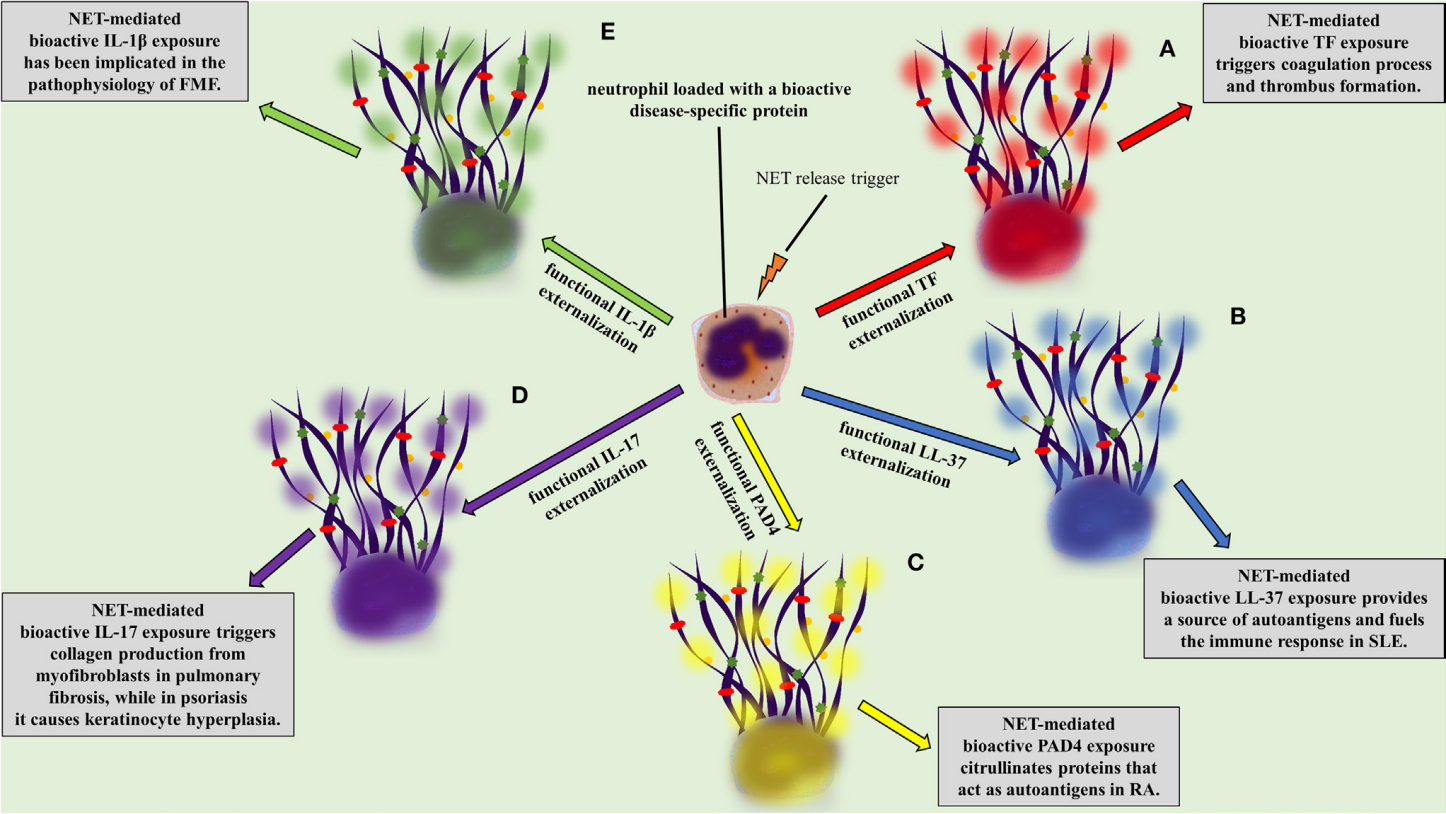
**NETopathies are ruled by the bioactive disease-related neutrophil extracellular trap (NET) proteins**. The clinical manifestations of **(A)** thrombosis, **(B)** systemic lupus erythematosus, **(C)** rheumatoid athritis (RA), **(D)** pulmonary fibrosis and psoriasis, **(E)** familiar Mediterranean fever are determined by the NET-mediated exposure of bioactive disease-related proteins.

There is evidence supporting the involvement of NETs in the pathophysiology of SLE. It has been shown that NETs are directly associated with the severity and the progression of the disease (91–95). Neutrophils from SLE patients are primed to undergo NET release (17, 96). Autoantibodies and more specifically antibodies against LL-37 have been shown to activate neutrophils for NET release (18, 19). On the other hand, NETs are composed of DNA, histones, and proteins-like LL-37, providing a possible source of autoantigens for the development of lupus-specific autoantibodies (Figure 2B) (17–19, 97–99). Interestingly, Villanueva et al. reported a neutrophil subpopulation in SLE, termed as low-density granulocytes (LDG), prone to release NETs, which promote vascular damage (18, 91, 100). It was further demonstrated that LL-37-bearing NETs fuel the immune response in SLE by activating plasmacytoid dendritic cells (pDCs) in an Immunoglobulin-Fc region receptor II-a (FcRIIa) and TLR9-dependent manner. This leads to interferon alpha (IFNα) production, which is a critical player in the pathogenesis of SLE. Furthermore, IFNα triggers NET generation and activates T and B cells leading to the production of antibodies against NETs, creating a vicious cycle (19, 97, 101, 102).

Interestingly, there is a disease-associated defect in the clearance of NETs, due to the reduced activity of DNase I and the increased amounts of DNase I inhibitors (17, 20, 94, 103–106), supporting the hypothesis that dysregulation of NET clearance may be one of the initial steps that lead to lupus-specific autoantibody production.

#### ANCA-Associated Vasculitis

Antineutrophil cytoplasmic antibody-associated vasculitis is described as a group of autoimmune diseases, characterized by the presence of autoantibodies against the neutrophil granule proteins, such as proteinase 3 (PR3) and MPO. The study by Kessenbrock et al. provided the initial evidence for the link between NETs and AAV. In this study, the intraglomerular deposition of NETs in biopsies from patients with small-vessel vasculitis was shown. Additionally, it was shown that neutrophils release NETs when activated with ANCA (107). Further studies confirmed the deposition of NETs in affected tissues from patients with AAV (14, 61, 108–110), whereas increased levels of circulating NET remnants were observed in patients with AAV (14, 22). Additionally, a recent study correlated AAV disease activity with the presence of NET-prone LDGs in peripheral blood (110). NETs were further associated with the AAV hypercoagulability, since NETs released during active disease are loaded with TF [Figure 2A (14, 111)].

Since PR3 and MPO are abundantly present in NETs, it has been proposed that NETs mediate the extracellular exposure of these potential autoantigens, having an important role in the initiation of the disease (17, 20, 21, 107). Sangaletti et al. have shown that myeloid DCs can acquire neutrophil proteins released in the form of NETs. Furthermore, immunization of mice with DCs co-cultured with NET remnants resulted in the development of MPO-ANCA and renal vasculitis (112). A common characteristic between SLE and AAV is the decreased degradation of NETs, attributed to the reduced activity and inhibition of DNase I, as well as to the protection over NETs by autoantibodies and components of the complement (17, 20, 107).

#### RA and Psoriasis

Rheumatoid arthritis is a chronic autoimmune disease that affects synovial joints. It is known that neutrophils are the most abundant cell type of synovial fluid in RA patients (113).

Recent studies identified the presence of NETs in the circulation and the release of NETs by synovial neutrophils (114, 115). Khandpur et al. have shown that TNF, IL-17, and anti-citrullinated protein antibodies (ACPA) promote NET release by neutrophils from patients with RA, whereas therapeutic blockade of TNF function has been shown to decrease the extensive NET generation that characterizes RA patients. Of interest, IL-17 was able to promote NET release only in neutrophils from patients with RA, which implies that the disease-specific inflammatory microenvironment primes neutrophils for NET formation (115).

Recent studies highlight that citrullinated histones in NETs consist autoantigens that stimulate and participate in the outset of the excessive inflammation, and more specifically in ACPA immune response, in RA (18, 115). It has been further demonstrated that RA-driven NETs are decorated with enzymatically active PAD4, which possibly further citrullinates targets, rendering them autoantigens (Figure 2C) (49, 67, 116). Finally, NETs in RA indirectly participate in the stimulation of distinct cell types, such as fibroblast-like synoviocytes, which invade and damage cartilage in RA (115, 117).

The possible involvement of NETs in the pathogenesis of psoriasis has been also proposed. Psoriasis is an autoimmune skin disorder characterized by epidermal hyperplasia and neutrophil infiltration in the epidermis. Neutrophils are involved in the pathophysiology of psoriasis, linking innate and adaptive immune system, and acting as a main source of IL-17 (66, 118, 119).

Interleukin 17 has a significant role in the pathophysiology of psoriasis causing keratinocyte hyperplasia (119, 120), whereas therapeutic administration of antibodies against IL-17 is efficacious in the treatment for psoriasis (121–123). The externalization of IL-17 in a bioactive form is feasible through NET formation (Figure 2D) (66, 124), which has been also observed in models of RA (115) and pancreatitis (125). The fact that the active form of IL-17 lies on NETs renders it an easily accessible target.

Taken together, a significant amount of evidence suggests that NETs contribute in the pathogenesis of several autoimmune disorders, acting either at the initiation of disease, providing a source of autoantigens, or promoting tissue injury (66, 90, 93, 107, 109, 115). There are reports suggesting that NETs can activate other inflammatory cell populations and promote the activation of the adaptive immune system (97, 102, 115). However, whether the specific structure of NETs and the possible modification in proteins loaded on NETs have a major impact in the break of tolerance and induction of autoimmunity still remains elusive.

### NETs in Autoinflammatory Diseases

Recent studies revealed a possible role for NETs in the inflammatory response that governs autoinflammatory syndromes, including gout and FMF.

Gout is an autoinflammatory type of arthritis caused by the intra-articular deposition of monosodium urate crystals (MSU crystals). The deposition causes inflammatory attacks due to innate immunity activation (126–129). Additionally, the chronic form of the disease is characterized by tophus formation, causing mechanical destruction of the joint (130). It has been shown that MSU crystals cause a strong induction of NETs (24, 131) which, in high neutrophil concentrations, ameliorates MSU crystal-induced inflammation by promoting the degradation of inflammatory cytokines and chemokines in a mouse model of MSU-induced inflammation (69, 132). Despite their protective role, NETs indirectly engender the destruction of the joint by easing the packing of MSU crystals and the formation of tophi (69, 132). However, whether NETs support the initiation of gouty inflammation in humans remains unanswered.

Familiar Mediterranean fever is a hereditary autoinflammatory disorder, characterized by inflammatory attacks and neutrophil infiltration into the affected sites (23). Moreover, it is an IL-1β-mediated disease, and this is clear due to the fact that IL-1β blockade constitutes an emerging treatment in FMF (23, 133, 134). During FMF attacks, neutrophils undergo excessive NET formation, which decreases after the inflammation dissolution (23).

During FMF attacks increased levels of circulating MPO–DNA complexes are detected, suggesting the release of NETs in the systemic circulation, whereas their levels normalize during the resolution phase of the disease (23). The detection of bioactive IL-1β in NETs released *ex vivo* by patient neutrophils or control neutrophils treated with FMF attack serum implies that neutrophils serve as critical effector cells in the amplification of inflammation in FMF (Figure 2E) (23).

### NETs in Metabolic Disorders

In type II diabetes (T2D), immunological changes lead to altered levels of cytokines and changes in both number and activation status of various leukocytes, including neutrophils (135). Until recently, it was thought that inflammatory responses may have a dual role in T2D, as they seem to have a causal relationship leading to resistance to insulin, while on the other hand they seem to be intensified by the hyperglycemic state, resulting in T2D complications (135).

Bearing in mind that diabetes affects neutrophil count and activity, that hyperglycemia-driven oxidative stress facilitates diabetic complications, and that neutrophils generate oxidative stress in diabetes, it was assumed that a dysregulation in NETosis may represent the link among hyperglycemia, oxidative stress, inflammation, and diabetic complications (27). In this direction, a recent study demonstrated that high glucose *in vitro* and hyperglycemia *in vivo* induce release of NETs and their products (27). Another study provided evidence that hyperglycemic conditions lead to the formation of short-lived and unstable NETs, while also prime neutrophils and constitutively activate NET formation, leading to reduced response to subsequent external stimuli (136). Thus, it was hypothesized that neutrophils primed due to hyperglycemia may not respond to further external stimulus in T2D patients, making them susceptible to infections (136). Finally, a third study demonstrates that, in T2D patients, dysregulated NET release caused by hyperglycemia is responsible for impairment of wound healing as well as for diabetic complications (137). Even though these studies support a role for NETs in T2D, it is not clear to what extent manipulation of neutrophils could ameliorate or prevent diabetic complications.

Moreover, there is evidence that neutrophils and NETs have a potential role in the pathogenesis of type I diabetes (28, 138, 139); however, their implication in the onset and/or the development of this disease has not been investigated so far.

### NETs in Lung Diseases and Fibrosis

Neutrophil extracellular traps have been implicated in inflammatory lung diseases and inflammatory-derived fibrosis (33). Several inflammatory lung diseases are characterized by the migration and detection of neutrophils and monocytes in the airway lumen and the bronchoalveolar lavage fluid (140). NETs have been associated with inflammatory diseases, such as chronic obstructive pulmonary disease (COPD), cystic fibrosis (CF), acute lung injury, acute respiratory distress syndrome, and asthma (29, 30).

Cystic fibrosis is characterized by abundant free DNA structures in airway fluids that increase the viscosity of the sputum and lead to airflow obstruction and tissue damage. Free DNA originates mainly from NETs released from neutrophils that are recruited to the area in an effort to kill the bacterial burden, but they finally contribute to the damage of lung tissue (31, 32). Additionally, it has been proved that NE plays an equally important role in CF, leading to tissue damage, especially in patients under treatment that are characterized by increased DNA cleavage (141). Recombinant human deoxyribonuclease (rhDNase) is an adjunctive to antibiotics treatment for patients with CF over the last two decades, showing a beneficial effect at least in a subpopulation of patients with CF (142, 143). Moreover, it has been reported that DNase I and histone-blocking antibodies have been used in mice against transfusion-related acute lung injury, in which NETs play a crucial role (144). Inhibition of either NE or NET release in general could be a novel future therapeutic strategy in patients with CF (141, 145).

There is evidence that the inflammatory microenvironment developed in chronic lung diseases including COPD and interstitial lung disease contributes either to localized or to generalized fibrosis, respectively. Specific fibrosis-related agents, such as cigarette smoke, magnesium silicate, and bleomycin, stimulate neutrophils to undergo NETosis. NETs indirectly regulate fibrosis by activating lung fibroblasts and differentiating them into myofibroblasts, through autophagy and histone hypercitrullination. Subsequently, NET remnants, such as IL-17, regulate connective tissue growth factor (CCN2) expression and collagen production by the differentiated fibroblasts and not their differentiation (Figure 2D). However, NET degradation significantly restricts these effects, indicating that it could be possibly used as a restraining mechanism against fibrosis (33).

### NETs in Cancer

In the last few years, NETs have redefined the role of neutrophils in tumor biology (34–36, 146–150). It is suggested that NETs may act within the primary tumor promoting tumor progression (146–148), while at remote sites they might sequester circulating cancer cells favoring metastasis (35, 36, 149). Additionally, NETs have been implicated in cancer-associated thrombosis (34, 147).

There is increasing evidence supporting that, in both experimental models and cancer patients, NET deposition in the tumor mass is associated with tumor progression (35, 146, 150–153). A finding that supports the implication of NETs in tumor biology is that tumor cells predispose neutrophils to undergo NETosis (34, 146). Moreover, in the tumor microenvironment, NETs interact with tumor cells and expose them to bioactive proteins, possibly favoring their survival through induction of proliferation and inhibition of apoptosis, as well as supporting their escape from the primary tumor (148).

Excessive NET deposition leads to a persistent inflammatory state (154–156), which in cancer probably promotes the expression of adhesion molecules (157–159). Under inflammatory conditions, when NET formation is induced, circulating tumor cells are more prone to adhere to end organ vasculature (158–160). Thus, given that the entrapment of bacteria is one of the primary roles attributed to NETs, they probably act accordingly to capture circulating tumor cells. By entrapping tumor cells and exposing them to various neutrophil-derived factors, NETs may generate a microenvironment rich in proteins and enzymes that promote tumor cell survival and progression (35, 36, 149, 153). Taken together, these data support a potential pro-metastatic role for NETs, involved in early adhesion, proliferation, invasion, and angiogenesis.

Neutrophil extracellular traps have also been implicated in cancer-associated thrombosis, the second most common cause of death in cancer patients (34). Recently, it was demonstrated that, through the generation of NETs, neutrophils provide a scaffold and a stimulus for platelet adhesion and thrombus formation (75). NETs were shown to promote coagulation as well (68, 75). Moreover, a recent study based on murine models reported that both leukemia and solid tumors produce a factor, G-CSF, that primes neutrophils to undergo NETosis and predisposes the host to thrombosis (34). In conclusion, NETs have been identified as a key player in cancer-associated thrombosis.

The biological significance of NETs in cancer remains unclear. It is hypothesized that initially they represent a reaction of the tumor environment against the growing cancer. However, NETs seem to play an adverse role in tumor growth, offering a scaffold with an array of biologically active molecules attached on it, which may promote malignant cell survival, growth, and local tumor expansion.

## Therapeutic and Diagnostic/Prognostic Potential of NETs

To date, clinical and experimental evidence highlight the significant role of NETs in the pathophysiology of the aforementioned diseases. Even though studies in animal models have shown the beneficial role of NET inhibition, especially in thrombosis, it is yet unknown whether NET-targeting therapies could be effective in clinic (161). NET induction or inhibition could be beneficial for patients with diseases that have been associated with restricted or excessive NET formation, respectively (Table 1). To this end, drug repositioning offers the opportunity for the immediate use of therapeutic agents that induce or inhibit NETs, which are already used in clinic (11).

**Table 1:** **Potential and applied therapeutic strategies targeting neutrophil extracellular traps (NETs)**.

NET formation blockade		NET integrity dismantling		NET components antagonism	

Drug (*activity*)	Disorder (*species*)	Drug (*activity*)	Disorder (*species*)	Drug (*activity*)	Disorder (*species*)
Hydroxychloroquine (*autophagy inhibition*)	SLE (*h*) (162)	DNases (*DNA dismantling*)	Thrombosis, cystic fibrosis (*h*) (64, 76, 142)	Secukinumab (*IL-17 inhibition*)	Psoriasis (*h*) (121)
N-acetylcysteine (*ROS reduction*)	SLE (*h*) (163)	Heparin (*chromatin dismantling*)	Thrombosis (*h*) (75)	Anakinra & Canakinumab (*IL-1*β *inhibition*)	FMF, gout (*h*) (23, 24, 134)
Sifalimumab (*IFN-*α *inhibition*)	SLE (*h*) (164)				
CI-Amidine (*PAD family inhibition*)	RA, SLE (*m*) (165, 166)				
GSK199 (*PAD4 inhibition*)	(*m*) (167)				
Adalimumab (*TNF inhibition*)	RA, psoriasis (*h*) (115, 168)				
Roflumilast (*neutrophil–platelet interaction inhibition*)	Thrombosis (*h*) (169)				
Eculizumab (*C5a inhibition*)	PNH (*h*) (170)				

*FMF, familial Mediterranean fever; h, human model; IFN-α, interferon alpha; IL-17, interleukin 17; IL-1β, interleukin 1 beta; m, murine model; PAD4, peptidyl arginine deiminase type IV; PNH, paroxysmal nocturnal hemoglobinuria; RA, rheumatoid arthritis; ROS, reactive oxygen species; SLE, systemic lupus erythematosus; TNF, tumor necrosis factor*.

**Figure 3:**
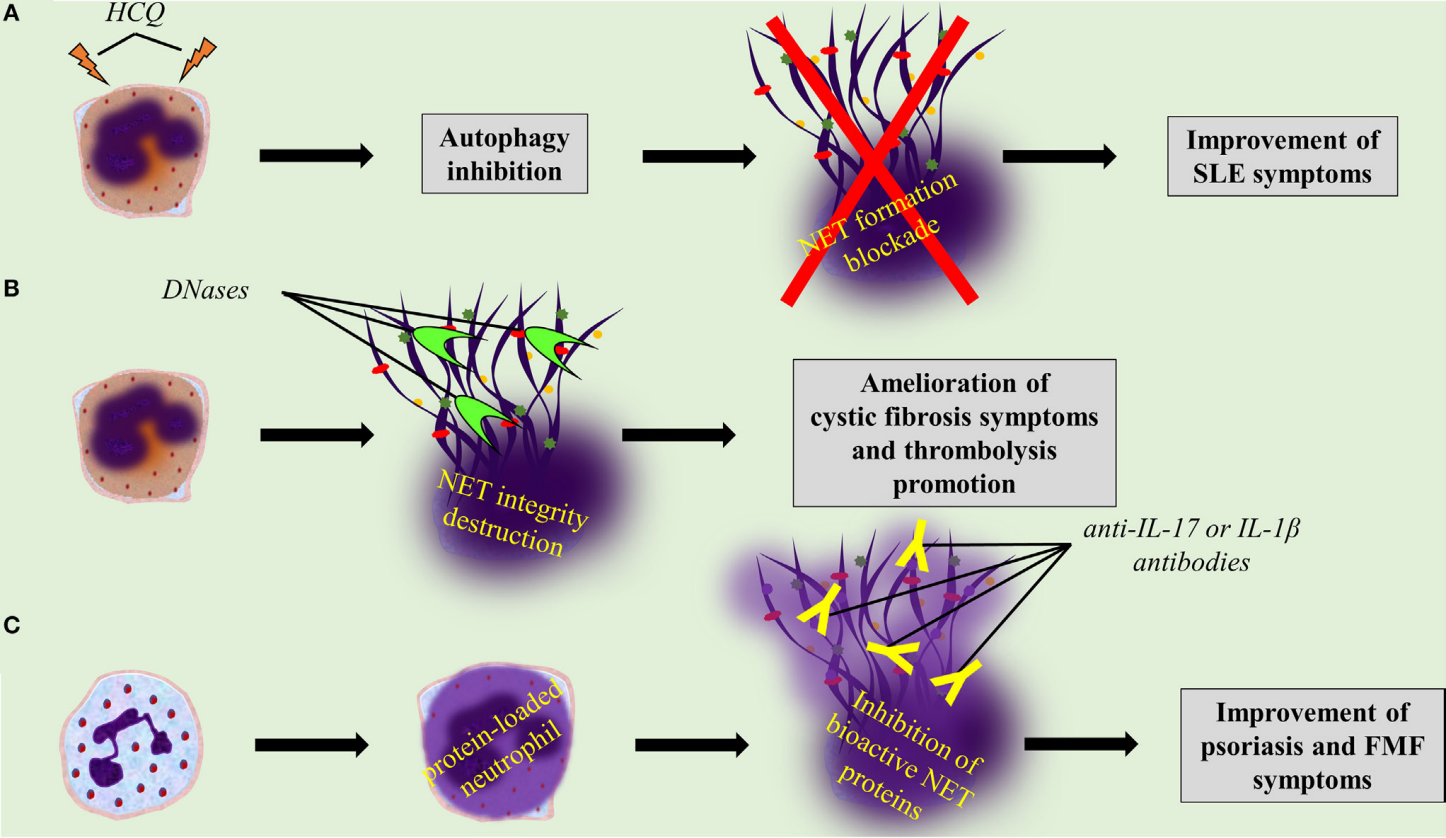
**Targeting neutrophil extracellular trap (NET) formation or integrity, or specific NET proteins, promises novel therapeutic strategies**. **(A)** Hydroxychloroquine inhibits NET formation through its anti-autophagic activity. **(B)** rhDNase and DNase I dismantle NET structures. **(C)** Anti-interleukin 17 (IL-17) and anti-interleukin 1 beta (IL-1β) antibodies blockade bioactive IL-17 and IL-1β on NETs, respectively.

Several drugs already used in clinical practice might affect either NET formation or integrity, or the expression of NET proteins. For instance, it is known that hydroxychloroquine (HCQ), a drug that has been used for decades in the treatment of SLE, has anti-autophagic effect (162). Since the autophagic machinery is an essential step for NETosis, the effectiveness of HCQ may be mediated through the indirect inhibition of NET formation (Table 1; Figure 3A). In addition, rhDNase administration, a therapy used in patients with CF aiming to the liquefaction of mucus (142), may possibly target NET structures. DNase promotes thrombolysis *via* degradation of NETs in murine models (Table 1; Figure 3B) (64, 76). Moreover, monoclonal antibodies are widely used against bioactive NET proteins, externalized through NET formation. In psoriasis, treatment with anti-IL-17 antibodies (121), probably targets the IL-17-decorated NETs, the main origin of bioactive IL-17 in psoriasis (66). Finally, NET-bound IL-1β may be one of the targets of anti-IL-1β therapies, such as canakinumab which targets bioactive IL-1β in FMF or gout patients (Table 1; Figure 3C) (134).

There are a few recent studies demonstrating that NETs could also have prognostic and/or diagnostic potential, as they could represent a disease activity marker for some of the aforementioned diseases (161). Furthermore, the measurement of NET release or specific NET protein expression in blood samples and biopsies could be a useful diagnostic tool (150, 171). Nevertheless, further experimental data are needed to evaluate the therapeutic, prognostic, and/or diagnostic potential of NETs.

## Conclusion

The identification of NETs and the characterization of their role in disease have revived the overlooked role of neutrophils in disease pathogenesis. Phagocytosis of pathogens and limitation of infection was considered the exclusive role of neutrophils. However, mechanistic studies in animal models and clinical observation dramatically altered our perception of the involvement of neutrophils in disease during the last decade. From a patrolling police force, neutrophils are considered nowadays an important player in autoimmune diseases or thrombotic disorders, which were previously thought to be exclusively mediated by adaptive immune system and platelet or endothelial cells, respectively. The characterization of the differential protein load and function of neutrophils, and subsequently of NETs, in distinct disorders can provide novel diagnostic targets and targets for therapeutic intervention. Additionally, the study on the role of NETs in modulation of tissue homeostasis, including the initiation and resolution of inflammation and the elucidation of the effect of NETs on different cell population involved in inflammatory, autoimmune, or thrombotic disorders, will increase our knowledge in the mechanisms that govern the pathogenesis of complex disorders. The clarification of the role of NETs in the pathogenesis of such disorders and the clinical use of therapeutic agents that target NETs will enable the identification of a group of disorders that could be characterized by the term NET-associated diseases or NETopathies.

## Author Contributions

AM, AA, SA, IM, and KR wrote the manuscript and created the figures. IM and KR also revised the manuscript.

## Conflict of Interest Statement

The authors declare that the research was conducted in the absence of any commercial or financial relationships that could be construed as a potential conflict of interest.

## References

[1] Fuchs TA Abed U Goosmann C Hurwitz R Schulze I Wahn V Novel cell death program leads to neutrophil extracellular traps. J Cell Biol (2007) 176(2):231–41. 10.1083/jcb.200606027 17210947 PMC2063942

[2] Papayannopoulos V Zychlinsky A. NETs: a new strategy for using old weapons. Trends Immunol (2009) 30(11):513–21. 10.1016/j.it.2009.07.011 19699684

[3] Nauseef WM Borregaard N. Neutrophils at work. Nat Immunol (2014) 15(7):602–11. 10.1038/ni.2921 24940954

[4] Mitroulis I Alexaki VI Kourtzelis I Ziogas A Hajishengallis G Chavakis T. Leukocyte integrins: role in leukocyte recruitment and as therapeutic targets in inflammatory disease. Pharmacol Ther (2015) 147:123–35. 10.1016/j.pharmthera.2014.11.008 25448040 PMC4324083

[5] Boettcher S Manz MG. Sensing and translation of pathogen signals into demand-adapted myelopoiesis. Curr Opin Hematol (2016) 23(1):5–10. 10.1097/MOH.0000000000000201 26554891

[6] Brinkmann V Reichard U Goosmann C Fauler B Uhlemann Y Weiss DS Neutrophil extracellular traps kill bacteria. Science (2004) 303(5663):1532–5. 10.1126/science.1092385 15001782

[7] Remijsen Q Kuijpers TW Wirawan E Lippens S Vandenabeele P Vanden Berghe T. Dying for a cause: NETosis, mechanisms behind an antimicrobial cell death modality. Cell Death Differ (2011) 18(4):581–8. 10.1038/cdd.2011.1 21293492 PMC3131909

[8] Beiter K Wartha F Albiger B Normark S Zychlinsky A Henriques-Normark B. An endonuclease allows *Streptococcus pneumoniae* to escape from neutrophil extracellular traps. Curr Biol (2006) 16(4):401–7. 10.1016/j.cub.2006.01.056 16488875

[9] Ramos-Kichik V Mondragón-Flores R Mondragón-Castelán M Gonzalez-Pozos S Muñiz-Hernandez S Rojas-Espinosa O Neutrophil extracellular traps are induced by *Mycobacterium tuberculosis*. Tuberculosis (Edinb) (2009) 89(1):29–37. 10.1016/j.tube.2008.09.009 19056316

[10] Urban CF Reichard U Brinkmann V Zychlinsky A. Neutrophil extracellular traps capture and kill *Candida albicans* yeast and hyphal forms. Cell Microbiol (2006) 8(4):668–76. 10.1111/j.1462-5822.2005.00659.x 16548892

[11] Konstantinidis T Kambas K Mitsios A Panopoulou M Tsironidou V Dellaporta E Immunomodulatory role of clarithromycin in *Acinetobacter baumannii* infection via formation of neutrophil extracellular traps. Antimicrob Agents Chemother (2016) 60(2):1040–8. 10.1128/AAC.02063-15 26643338 PMC4750671

[12] Kambas K Mitroulis I Apostolidou E Girod A Chrysanthopoulou A Pneumatikos I Autophagy mediates the delivery of thrombogenic tissue factor to neutrophil extracellular traps in human sepsis. PLoS One (2012) 7(9):e45427. 10.1371/journal.pone.0045427 23029002 PMC3446899

[13] Kambas K Mitroulis I Ritis K. The emerging role of neutrophils in thrombosis-the journey of TF through NETs. Front Immunol (2012) 3:385. 10.3389/fimmu.2012.00385 23264778 PMC3524512

[14] Kambas K Chrysanthopoulou A Vassilopoulos D Apostolidou E Skendros P Girod A Tissue factor expression in neutrophil extracellular traps and neutrophil derived microparticles in antineutrophil cytoplasmic antibody associated vasculitis may promote thromboinflammation and the thrombophilic state associated with the disease. Ann Rheum Dis (2014) 73(10):1854–63. 10.1136/annrheumdis-2013-203430 23873874

[15] de Boer OJ Li X Teeling P Mackaay C Ploegmakers HJ van der Loos CM Neutrophils, neutrophil extracellular traps and interleukin-17 associate with the organisation of thrombi in acute myocardial infarction. Thromb Haemost (2013) 109(2):290–7. 10.1160/TH12-06-0425 23238559

[16] Stakos DA Kambas K Konstantinidis T Mitroulis I Apostolidou E Arelaki S Expression of functional tissue factor by neutrophil extracellular traps in culprit artery of acute myocardial infarction. Eur Heart J (2015) 36(22):1405–14. 10.1093/eurheartj/ehv007 25660055 PMC4458286

[17] Hakkim A Fürnrohr BG Amann K Laube B Abed UA Brinkmann V Impairment of neutrophil extracellular trap degradation is associated with lupus nephritis. Proc Natl Acad Sci U S A (2010) 107(21):9813–8. 10.1073/pnas.0909927107 20439745 PMC2906830

[18] Lande R Ganguly D Facchinetti V Frasca L Conrad C Gregorio J Neutrophils activate plasmacytoid dendritic cells by releasing self-DNA-peptide complexes in systemic lupus erythematosus. Sci Transl Med (2011) 3(73):73ra19. 10.1126/scitranslmed.3001180 21389263 PMC3399524

[19] Kahlenberg JM Kaplan MJ. Little peptide, big effects: the role of LL-37 in inflammation and autoimmune disease. J Immunol (2013) 191(10):4895–901. 10.4049/jimmunol.1302005 24185823 PMC3836506

[20] Leffler J Martin M Gullstrand B Tydén H Lood C Truedsson L Neutrophil extracellular traps that are not degraded in systemic lupus erythematosus activate complement exacerbating the disease. J Immunol (2012) 188(7):3522–31. 10.4049/jimmunol.1102404 22345666

[21] Couser WG Johnson RJ. What is myeloperoxidase doing in ANCA-associated glomerulonephritis? Kidney Int (2015) 88(5):938–40. 10.1038/ki.2015.259 26579678

[22] Söderberg D Kurz T Motamedi A Hellmark T Eriksson P Segelmark M. Increased levels of neutrophil extracellular trap remnants in the circulation of patients with small vessel vasculitis, but an inverse correlation to anti-neutrophil cytoplasmic antibodies during remission. Rheumatology (Oxford) (2015) 54(11):2085–94. 10.1093/rheumatology/kev217 26170375

[23] Apostolidou E Skendros P Kambas K Mitroulis I Konstantinidis T Chrysanthopoulou A Neutrophil extracellular traps regulate IL-1β-mediated inflammation in familial Mediterranean fever. Ann Rheum Dis (2016) 75(1):269–77. 10.1136/annrheumdis-2014-205958 25261578

[24] Mitroulis I Kambas K Chrysanthopoulou A Skendros P Apostolidou E Kourtzelis I Neutrophil extracellular trap formation is associated with IL-1β and autophagy-related signaling in gout. PLoS One (2011) 6(12):e29318. 10.1371/journal.pone.0029318 22195044 PMC3241704

[25] Bennike TB Carlsen TG Ellingsen T Bonderup OK Glerup H Bøgsted M Neutrophil extracellular traps in ulcerative colitis: a proteome analysis of intestinal biopsies. Inflamm Bowel Dis (2015) 21(9):2052–67. 10.1097/MIB.0000000000000460 25993694 PMC4603666

[26] He Z Si Y Jiang T Ma R Zhang Y Cao M Phosphotidylserine exposure and neutrophil extracellular traps enhance procoagulant activity in patients with inflammatory bowel disease. Thromb Haemost (2016) 115(4):738–51. 10.1160/TH15-09-0710 26660948

[27] Menegazzo L Ciciliot S Poncina N Mazzucato M Persano M Bonora B NETosis is induced by high glucose and associated with type 2 diabetes. Acta Diabetol (2015) 52(3):497–503. 10.1007/s00592-014-0676-x 25387570

[28] Wang Y Xiao Y Zhong L Ye D Zhang J Tu Y Increased neutrophil elastase and proteinase 3 and augmented NETosis are closely associated with β-cell autoimmunity in patients with type 1 diabetes. Diabetes (2014) 63(12):4239–48. 10.2337/db14-0480 25092677

[29] Cheng OZ Palaniyar N. NET balancing: a problem in inflammatory lung diseases. Front Immunol (2013) 4:1. 10.3389/fimmu.2013.00001 23355837 PMC3553399

[30] Grabcanovic-Musija F Obermayer A Stoiber W Krautgartner W-D Steinbacher P Winterberg N Neutrophil extracellular trap (NET) formation characterises stable and exacerbated COPD and correlates with airflow limitation. Respir Res (2015) 16:59. 10.1186/s12931-015-0221-7 25994149 PMC4455316

[31] Marcos V Zhou-Suckow Z Önder Yildirim A Bohla A Hector A Vitkov L Free DNA in cystic fibrosis airway fluids correlates with airflow obstruction. Mediators Inflamm (2015) 2015:408935. 10.1155/2015/408935 25918476 PMC4397025

[32] Dwyer M Shan Q D’Ortona S Maurer R Mitchell R Olesen H Cystic fibrosis sputum DNA has NETosis characteristics and neutrophil extracellular trap release is regulated by macrophage migration-inhibitory factor. J Innate Immun (2014) 6(6):765–79. 10.1159/000363242 24862346 PMC4201867

[33] Chrysanthopoulou A Mitroulis I Apostolidou E Arelaki S Mikroulis D Konstantinidis T Neutrophil extracellular traps promote differentiation and function of fibroblasts. J Pathol (2014) 233(3):294–307. 10.1002/path.4359 24740698

[34] Demers M Krause DS Schatzberg D Martinod K Voorhees JR Fuchs TA Cancers predispose neutrophils to release extracellular DNA traps that contribute to cancer-associated thrombosis. Proc Natl Acad Sci U S A (2012) 109(32):13076–81. 10.1073/pnas.1200419109 22826226 PMC3420209

[35] Cools-Lartigue J Spicer J McDonald B Gowing S Chow S Giannias B Neutrophil extracellular traps sequester circulating tumor cells and promote metastasis. J Clin Invest (2013) 123(8):3446–58. 10.1172/JCI67484 23863628 PMC3726160

[36] Tohme S Yazdani HO Al-Khafaji AB Chidi AP Loughran P Mowen K Neutrophil extracellular traps promote the development and progression of liver metastases after surgical stress. Cancer Res (2016) 76(6):1367–80. 10.1158/0008-5472.CAN-15-1591 26759232 PMC4794393

[37] Yang H Biermann MH Brauner JM Liu Y Zhao Y Herrmann M. New insights into neutrophil extracellular traps: mechanisms of formation and role in inflammation. Front Immunol (2016) 7:302. 10.3389/fimmu.2016.00302 27570525 PMC4981595

[38] Lood C Blanco LP Purmalek MM Carmona-Rivera C De Ravin SS Smith CK Neutrophil extracellular traps enriched in oxidized mitochondrial DNA are interferogenic and contribute to lupus-like disease. Nat Med (2016) 22(2):146–53. 10.1038/nm.4027 26779811 PMC4742415

[39] Yousefi S Mihalache C Kozlowski E Schmid I Simon HU. Viable neutrophils release mitochondrial DNA to form neutrophil extracellular traps. Cell Death Differ (2009) 16(11):1438–44. 10.1038/cdd.2009.96 19609275

[40] Steinberg BE Grinstein S. Unconventional roles of the NADPH oxidase: signaling, ion homeostasis, and cell death. Sci STKE (2007) 2007(379):e11. 10.1126/stke.3792007pe11 17392241

[41] Yipp BG Petri B Salina D Jenne CN Scott BNV Zbytnuik LD Infection-induced NETosis is a dynamic process involving neutrophil multitasking in vivo. Nat Med (2012) 18(9):1386–93. 10.1038/nm.2847 22922410 PMC4529131

[42] Yipp BG Kubes P. NETosis: how vital is it? Blood (2013) 122(16):2784–94. 10.1182/blood-2013-04-457671 24009232

[43] Wang Y Li M Stadler S Correll S Li P Wang D Histone hypercitrullination mediates chromatin decondensation and neutrophil extracellular trap formation. J Cell Biol (2009) 184(2):205–13. 10.1083/jcb.200806072 19153223 PMC2654299

[44] Papayannopoulos V Metzler KD Hakkim A Zychlinsky A. Neutrophil elastase and myeloperoxidase regulate the formation of neutrophil extracellular traps. J Cell Biol (2010) 191(3):677–91. 10.1083/jcb.201006052 20974816 PMC3003309

[45] Brinkmann V Goosmann C Kühn LI Zychlinsky A Automatic quantification of in vitro NET formation. Front Immunol (2013) 3:413 10.3389/fimmu.2012.00413 23316198 PMC3540390

[46] Zawrotniak M Rapala-Kozik M. Neutrophil extracellular traps (NETs) – formation and implications. Acta Biochim Pol (2013) 60(3):277–84. 23819131

[47] Kolaczkowska E Jenne CN Surewaard BGJ Thanabalasuriar A Lee W-Y Sanz M-J Molecular mechanisms of NET formation and degradation revealed by intravital imaging in the liver vasculature. Nat Commun (2015) 6:6673. 10.1038/ncomms7673 25809117 PMC4389265

[48] Kusunoki Y Nakazawa D Shida H Hattanda F Miyoshi A Masuda S Peptidylarginine deiminase inhibitor suppresses neutrophil extracellular trap formation and MPO-ANCA production. Front Immunol (2016) 7:227. 10.3389/fimmu.2016.00227 27375623 PMC4896908

[49] Li P Li M Lindberg MR Kennett MJ Xiong N Wang Y. PAD4 is essential for antibacterial innate immunity mediated by neutrophil extracellular traps. J Exp Med (2010) 207(9):1853–62. 10.1084/jem.20100239 20733033 PMC2931169

[50] Nauseef WM Kubes P. Pondering neutrophil extracellular traps with healthy skepticism. Cell Microbiol (2016) 18(10):1349–57. 10.1111/cmi.12652 27470975 PMC5025378

[51] O’Neill LAJ Kishton RJ Rathmell J. A guide to immunometabolism for immunologists. Nat Rev Immunol (2016) 16(9):553–65. 10.1038/nri.2016.70 27396447 PMC5001910

[52] Fossati G Moulding DA Spiller DG Moots RJ White MRH Edwards SW. The mitochondrial network of human neutrophils: role in chemotaxis, phagocytosis, respiratory burst activation, and commitment to apoptosis. J Immunol (2003) 170(4):1964–72. 10.4049/jimmunol.170.4.1964 12574365

[53] Rodríguez-Espinosa O Rojas-Espinosa O Moreno-Altamirano MMB López-Villegas EO Sánchez-García FJ. Metabolic requirements for neutrophil extracellular traps formation. Immunology (2015) 145(2):213–24. 10.1111/imm.12437 25545227 PMC4427386

[54] Azevedo EP Rochael NC Guimarães-Costa AB de Souza-Vieira TS Ganilho J Saraiva EM A metabolic shift toward pentose phosphate pathway is necessary for amyloid fibril- and phorbol 12-myristate 13-acetate-induced neutrophil extracellular trap (NET) formation. J Biol Chem (2015) 290(36):22174–83. 10.1074/jbc.M115.640094 26198639 PMC4571968

[55] Douda DN Khan MA Grasemann H Palaniyar N. SK3 channel and mitochondrial ROS mediate NADPH oxidase-independent NETosis induced by calcium influx. Proc Natl Acad Sci U S A (2015) 112(9):2817–22. 10.1073/pnas.1414055112 25730848 PMC4352781

[56] Remijsen Q Vanden Berghe T Wirawan E Asselbergh B Parthoens E De Rycke R Neutrophil extracellular trap cell death requires both autophagy and superoxide generation. Cell Res (2011) 21(2):290–304. 10.1038/cr.2010.150 21060338 PMC3193439

[57] Eisenberg-Lerner A Bialik S Simon H-U Kimchi A. Life and death partners: apoptosis, autophagy and the cross-talk between them. Cell Death Differ (2009) 16(7):966–75. 10.1038/cdd.2009.33 19325568

[58] Cuervo AM Autophagy: many paths to the same end. Mol Cell Biochem (2004) 263(1–2):55–72. 10.1023/B:MCBI.0000041848.57020.57 27520665

[59] Itakura A McCarty OJT. Pivotal role for the mTOR pathway in the formation of neutrophil extracellular traps via regulation of autophagy. Am J Physiol Cell Physiol (2013) 305(3):C348–54. 10.1152/ajpcell.00108.2013 23720022 PMC3742850

[60] Sha L-L Wang H Wang C Peng H-Y Chen M Zhao M-H. Autophagy is induced by anti-neutrophil cytoplasmic Abs and promotes neutrophil extracellular traps formation. Innate Immun (2016) 22(8):658–65. 10.1177/1753425916668981 27670946

[61] Tang S Zhang Y Yin S-W Gao X-J Shi W-W Wang Y Neutrophil extracellular trap formation is associated with autophagy-related signalling in ANCA-associated vasculitis. Clin Exp Immunol (2015) 180(3):408–18. 10.1111/cei.12589 25644394 PMC4449769

[62] Maugeri N Campana L Gavina M Covino C De Metrio M Panciroli C Activated platelets present high mobility group box 1 to neutrophils, inducing autophagy and promoting the extrusion of neutrophil extracellular traps. J Thromb Haemost (2014) 12(12):2074–88. 10.1111/jth.12710 25163512

[63] Ramirez GA Manfredi AA Rovere-Querini P Maugeri N Bet on NETs! Or on how to translate basic science into clinical practice. Front Immunol (2016) 7:417 10.3389/fimmu.2016.00417 27790216 PMC5063843

[64] von Brühl M-L Stark K Steinhart A Chandraratne S Konrad I Lorenz M Monocytes, neutrophils, and platelets cooperate to initiate and propagate venous thrombosis in mice in vivo. J Exp Med (2012) 209(4):819–35. 10.1084/jem.20112322 22451716 PMC3328366

[65] Kimball AS Obi AT Diaz JA Henke PK. The emerging role of NETs in venous thrombosis and immunothrombosis. Front Immunol (2016) 7:236. 10.3389/fimmu.2016.00236 27446071 PMC4921471

[66] Lin AM Rubin CJ Khandpur R Wang JY Riblett M Yalavarthi S Mast cells and neutrophils release IL-17 through extracellular trap formation in psoriasis. J Immunol (2011) 187(1):490–500. 10.4049/jimmunol.1100123 21606249 PMC3119764

[67] Reyes-Castillo Z Palafox-Sánchez CA Parra-Rojas I Martínez-Bonilla GE del Toro-Arreola S Ramírez-Dueñas MG Comparative analysis of autoantibodies targeting peptidylarginine deiminase type 4, mutated citrullinated vimentin and cyclic citrullinated peptides in rheumatoid arthritis: associations with cytokine profiles, clinical and genetic features. Clin Exp Immunol (2015) 182(2):119–31. 10.1111/cei.12677 26149185 PMC4608501

[68] Massberg S Grahl L von Bruehl M-L Manukyan D Pfeiler S Goosmann C Reciprocal coupling of coagulation and innate immunity via neutrophil serine proteases. Nat Med (2010) 16(8):887–96. 10.1038/nm.2184 20676107

[69] Schauer C Janko C Munoz LE Zhao Y Kienhöfer D Frey B Aggregated neutrophil extracellular traps limit inflammation by degrading cytokines and chemokines. Nat Med (2014) 20(5):511–7. 10.1038/nm.3547 24784231

[70] Savchenko AS Martinod K Seidman MA Wong SL Borissoff JI Piazza G Neutrophil extracellular traps form predominantly during the organizing stage of human venous thromboembolism development. J Thromb Haemost (2014) 12(6):860–70. 10.1111/jth.12571 24674135 PMC4055516

[71] Riegger J Byrne RA Joner M Chandraratne S Gershlick AH Ten Berg JM Histopathological evaluation of thrombus in patients presenting with stent thrombosis. A multicenter European study: a report of the prevention of late stent thrombosis by an interdisciplinary global European effort consortium. Eur Heart J (2016) 37(19):1538–49. 10.1093/eurheartj/ehv419 26761950 PMC4872283

[72] Clark SR Ma AC Tavener SA McDonald B Goodarzi Z Kelly MM Platelet TLR4 activates neutrophil extracellular traps to ensnare bacteria in septic blood. Nat Med (2007) 13(4):463–9. 10.1038/nm1565 17384648

[73] Delabranche X Stiel L Severac F Galoisy A-C Mauvieux L Zobairi F Evidence of NETosis in septic shock-induced disseminated intravascular coagulation. Shock (2016). 10.1097/SHK.0000000000000719 27488091

[74] Yalavarthi S Gould TJ Rao AN Mazza LF Morris AE Núñez-Álvarez C Release of neutrophil extracellular traps by neutrophils stimulated with antiphospholipid antibodies: a newly identified mechanism of thrombosis in the antiphospholipid syndrome. Arthritis Rheumatol (2015) 67(11):2990–3003. 10.1002/art.39247 26097119 PMC4626310

[75] Fuchs TA Brill A Duerschmied D Schatzberg D Monestier M Myers DD Extracellular DNA traps promote thrombosis. Proc Natl Acad Sci U S A (2010) 107(36):15880–5. 10.1073/pnas.1005743107 20798043 PMC2936604

[76] Brill A Fuchs TA Savchenko AS Thomas GM Martinod K De Meyer SF Neutrophil extracellular traps promote deep vein thrombosis in mice. J Thromb Haemost (2012) 10(1):136–44. 10.1111/j.1538-7836.2011.04544.x 22044575 PMC3319651

[77] Cedervall J Zhang Y Huang H Zhang L Femel J Dimberg A Neutrophil extracellular traps accumulate in peripheral blood vessels and compromise organ function in tumor-bearing animals. Cancer Res (2015) 75(13):2653–62. 10.1158/0008-5472.CAN-14-3299 26071254

[78] Nakazawa D Tomaru U Yamamoto C Jodo S Ishizu A. Abundant neutrophil extracellular traps in thrombus of patient with microscopic polyangiitis. Front Immunol (2012) 3:333. 10.3389/fimmu.2012.00333 23162551 PMC3495275

[79] van Montfoort ML Stephan F Lauw MN Hutten BA Van Mierlo GJ Solati S Circulating nucleosomes and neutrophil activation as risk factors for deep vein thrombosis. Arterioscler Thromb Vasc Biol (2013) 33(1):147–51. 10.1161/ATVBAHA.112.300498 23104849

[80] Diaz JA Fuchs TA Jackson TO Kremer Hovinga JA Lämmle B Henke PK Plasma DNA is elevated in patients with deep vein thrombosis. J Vasc Surg Venous Lymphat Disord (2013) 1(4):341–8.e1. 10.1016/j.jvsv.2012.12.002 24187669 PMC3810974

[81] van Bijnen STA Wouters D van Mierlo GJ Muus P Zeerleder S. Neutrophil activation and nucleosomes as markers of systemic inflammation in paroxysmal nocturnal hemoglobinuria: effects of eculizumab. J Thromb Haemost (2015) 13(11):2004–11. 10.1111/jth.13125 26333021

[82] Fuchs TA Kremer Hovinga JA Schatzberg D Wagner DD Lämmle B. Circulating DNA and myeloperoxidase indicate disease activity in patients with thrombotic microangiopathies. Blood (2012) 120(6):1157–64. 10.1182/blood-2012-02-412197 22611154 PMC3418712

[83] Lakbakbi S Debrumetz A Terryn C Szymezak J Rieu P Nguyen P. Tissue factor expressed by adherent cells contributes to hemodialysis-membrane thrombogenicity. Thromb Res (2016) 144:218–23. 10.1016/j.thromres.2016.05.017 27423005

[84] Stark K Philippi V Stockhausen S Busse J Antonelli A Miller M Disulfide HMGB1 derived from platelets coordinates venous thrombosis in mice. Blood (2016) 128(20):2435–49. 10.1182/blood-2016-04-710632 27574188 PMC5147023

[85] Martinod K Demers M Fuchs TA Wong SL Brill A Gallant M Neutrophil histone modification by peptidylarginine deiminase 4 is critical for deep vein thrombosis in mice. Proc Natl Acad Sci U S A (2013) 110(21):8674–9. 10.1073/pnas.1301059110 23650392 PMC3666755

[86] Meng H Yalavarthi S Kanthi Y Mazza LF Elfline MA Luke CE In vivo role of neutrophil extracellular traps in antiphospholipid antibody-mediated venous thrombosis. Arthritis Rheumatol (2016). 10.1002/art.39938 27696751 PMC5329054

[87] Rapaport SI Rao LV The tissue factor pathway: how it has become a “prima ballerina”. Thromb Haemost (1995) 74(1):7–17. 8578528

[88] Slaba I Wang J Kolaczkowska E McDonald B Lee W-Y Kubes P. Imaging the dynamic platelet-neutrophil response in sterile liver injury and repair in mice. Hepatology (2015) 62(5):1593–605. 10.1002/hep.28003 26202541

[89] Tsokos GC Systemic lupus erythematosus. N Engl J Med (2011) 365(22):2110–21. 10.1056/NEJMra1100359 22129255

[90] Knight JS Carmona-Rivera C Kaplan MJ. Proteins derived from neutrophil extracellular traps may serve as self-antigens and mediate organ damage in autoimmune diseases. Front Immunol (2012) 3:380. 10.3389/fimmu.2012.00380 23248629 PMC3521997

[91] Bosch X Systemic lupus erythematosus and the neutrophil. N Engl J Med (2011) 365(8):758–60. 10.1056/NEJMcibr1107085 21864171

[92] Smith CK Kaplan MJ. The role of neutrophils in the pathogenesis of systemic lupus erythematosus. Curr Opin Rheumatol (2015) 27(5):448–53. 10.1097/BOR.0000000000000197 26125102 PMC12351535

[93] Villanueva E Yalavarthi S Berthier CC Hodgin JB Khandpur R Lin AM Netting neutrophils induce endothelial damage, infiltrate tissues, and expose immunostimulatory molecules in systemic lupus erythematosus. J Immunol (2011) 187(1):538–52. 10.4049/jimmunol.1100450 21613614 PMC3119769

[94] Leffler J Gullstrand B Jönsen A Nilsson J-Å Martin M Blom AM Degradation of neutrophil extracellular traps co-varies with disease activity in patients with systemic lupus erythematosus. Arthritis Res Ther (2013) 15(4):R84. 10.1186/ar4264 23945056 PMC3978901

[95] Smith CK Vivekanandan-Giri A Tang C Knight JS Mathew A Padilla RL Neutrophil extracellular trap-derived enzymes oxidize high-density lipoprotein: an additional proatherogenic mechanism in systemic lupus erythematosus. Arthritis Rheumatol (2014) 66(9):2532–44. 10.1002/art.38703 24838349 PMC4146708

[96] Dieker J Tel J Pieterse E Thielen A Rother N Bakker M Circulating apoptotic microparticles in systemic lupus erythematosus patients drive the activation of dendritic cell subsets and prime neutrophils for NETosis. Arthritis Rheumatol (2016) 68(2):462–72. 10.1002/art.39417 26360137

[97] Garcia-Romo GS Caielli S Vega B Connolly J Allantaz F Xu Z Netting neutrophils are major inducers of type I IFN production in pediatric systemic lupus erythematosus. Sci Transl Med (2011) 3(73):73ra20. 10.1126/scitranslmed.3001201 21389264 PMC3143837

[98] Carmona-Rivera C Kaplan MJ. Detection of SLE antigens in neutrophil extracellular traps (NETs). Methods Mol Biol (2014) 1134:151–61. 10.1007/978-1-4939-0326-9_11 24497360 PMC4123114

[99] Pieterse E Hofstra J Berden J Herrmann M Dieker J van der Vlag J. Acetylated histones contribute to the immunostimulatory potential of neutrophil extracellular traps in systemic lupus erythematosus. Clin Exp Immunol (2015) 179(1):68–74. 10.1111/cei.12359 24758196 PMC4260898

[100] Carmona-Rivera C Zhao W Yalavarthi S Kaplan MJ. Neutrophil extracellular traps induce endothelial dysfunction in systemic lupus erythematosus through the activation of matrix metalloproteinase-2. Ann Rheum Dis (2015) 74(7):1417–24. 10.1136/annrheumdis-2013-204837 24570026 PMC4143484

[101] Yu Y Su K. Neutrophil extracellular traps and systemic lupus erythematosus. J Clin Cell Immunol (2013) 4(4):139. 10.4172/2155-9899.1000139 24244889 PMC3826916

[102] Lindau D Mussard J Rabsteyn A Ribon M Kötter I Igney A TLR9 independent interferon α production by neutrophils on NETosis in response to circulating chromatin, a key lupus autoantigen. Ann Rheum Dis (2014) 73(12):2199–207. 10.1136/annrheumdis-2012-203041 24013727

[103] Chitrabamrung S Rubin RL Tan EM. Serum deoxyribonuclease I and clinical activity in systemic lupus erythematosus. Rheumatol Int (1981) 1(2):55–60. 10.1007/BF00541153 6287560

[104] Yeh T-M Chang H-C Liang C-C Wu J-J Liu M-F. Deoxyribonuclease-inhibitory antibodies in systemic lupus erythematosus. J Biomed Sci (2003) 10(5):544–51. 10.1007/BF02256116 12928595

[105] Chauhan SK Rai R Singh VV Rai M Rai G. Differential clearance mechanisms, neutrophil extracellular trap degradation and phagocytosis, are operative in systemic lupus erythematosus patients with distinct autoantibody specificities. Immunol Lett (2015) 168(2):254–9. 10.1016/j.imlet.2015.09.016 26434792

[106] Leffler J Ciacma K Gullstrand B Bengtsson AA Martin M Blom AM. A subset of patients with systemic lupus erythematosus fails to degrade DNA from multiple clinically relevant sources. Arthritis Res Ther (2015) 17:205. 10.1186/s13075-015-0726-y 26268365 PMC4535789

[107] Kessenbrock K Krumbholz M Schönermarck U Back W Gross WL Werb Z Netting neutrophils in autoimmune small-vessel vasculitis. Nat Med (2009) 15(6):623–5. 10.1038/nm.1959 19448636 PMC2760083

[108] O’Sullivan KM Lo CY Summers SA Elgass KD McMillan PJ Longano A Renal participation of myeloperoxidase in antineutrophil cytoplasmic antibody (ANCA)-associated glomerulonephritis. Kidney Int (2015) 88(5):1030–46. 10.1038/ki.2015.202 26176828

[109] Yoshida M Sasaki M Sugisaki K Yamaguchi Y Yamada M. Neutrophil extracellular trap components in fibrinoid necrosis of the kidney with myeloperoxidase-ANCA-associated vasculitis. Clin Kidney J (2013) 6(3):308–12. 10.1093/ckj/sft048 26064491 PMC4400491

[110] Grayson PC Carmona-Rivera C Xu L Lim N Gao Z Asare AL Neutrophil-related gene expression and low-density granulocytes associated with disease activity and response to treatment in antineutrophil cytoplasmic antibody-associated vasculitis. Arthritis Rheumatol (2015) 67(7):1922–32. 10.1002/art.39153 25891759 PMC4485551

[111] Huang Y-M Wang H Wang C Chen M Zhao M-H. Promotion of hypercoagulability in antineutrophil cytoplasmic antibody-associated vasculitis by C5a-induced tissue factor-expressing microparticles and neutrophil extracellular traps. Arthritis Rheumatol (2015) 67(10):2780–90. 10.1002/art.39239 26097236

[112] Sangaletti S Tripodo C Chiodoni C Guarnotta C Cappetti B Casalini P Neutrophil extracellular traps mediate transfer of cytoplasmic neutrophil antigens to myeloid dendritic cells toward ANCA induction and associated autoimmunity. Blood (2012) 120(15):3007–18. 10.1182/blood-2012-03-416156 22932797

[113] Wright HL Moots RJ Edwards SW The multifactorial role of neutrophils in rheumatoid arthritis. Nat Rev Rheumatol (2014) 10(10):593–601. 10.1038/nrrheum.2014.80 24914698

[114] Deane KD Norris JM Holers VM Pre-clinical rheumatoid arthritis: identification, evaluation and future directions for investigation. Rheum Dis Clin North Am (2010) 36(2):213–41. 10.1016/j.rdc.2010.02.001 20510231 PMC2879710

[115] Khandpur R Carmona-Rivera C Vivekanandan-Giri A Gizinski A Yalavarthi S Knight JS NETs are a source of citrullinated autoantigens and stimulate inflammatory responses in rheumatoid arthritis. Sci Transl Med (2013) 5(178):178ra40. 10.1126/scitranslmed.3005580 23536012 PMC3727661

[116] Zhao J Zhao Y He J Jia R Li Z. Prevalence and significance of anti-peptidylarginine deiminase 4 antibodies in rheumatoid arthritis. J Rheumatol (2008) 35(6):969–74. 18398945

[117] Spengler J Lugonja B Ytterberg AJ Zubarev RA Creese AJ Pearson MJ Release of active peptidyl arginine deiminases by neutrophils can explain production of extracellular citrullinated autoantigens in rheumatoid arthritis synovial fluid. Arthritis Rheumatol (2015) 67(12):3135–45. 10.1002/art.39313 26245941 PMC4832324

[118] Nestle FO Kaplan DH Barker J Psoriasis. N Engl J Med (2009) 361(5):496–509. 10.1056/NEJMra0804595 19641206

[119] Schön MP Broekaert SMC Erpenbeck L. Sexy again: the renaissance of neutrophils in psoriasis. Exp Dermatol (2016). 10.1111/exd.13067 27194625

[120] Muhr P Renne J Schaefer V Werfel T Wittmann M. Primary human keratinocytes efficiently induce IL-1-dependent IL-17 in CCR6+ T cells. Exp Dermatol (2010) 19(12):1105–7. 10.1111/j.1600-0625.2010.01134.x 20812962

[121] Hueber W Patel DD Dryja T Wright AM Koroleva I Bruin G Effects of AIN457, a fully human antibody to interleukin-17A, on psoriasis, rheumatoid arthritis, and uveitis. Sci Transl Med (2010) 2(52):52ra72 10.1126/scitranslmed.3001107 20926833

[122] Leonardi C Matheson R Zachariae C Cameron G Li L Edson-Heredia E Anti-interleukin-17 monoclonal antibody ixekizumab in chronic plaque psoriasis. N Engl J Med (2012) 366(13):1190–9. 10.1056/NEJMoa1109997 22455413

[123] Papp KA Leonardi C Menter A Ortonne J-P Krueger JG Kricorian G Brodalumab, an anti-interleukin-17-receptor antibody for psoriasis. N Engl J Med (2012) 366(13):1181–9. 10.1056/NEJMoa1109017 22455412

[124] Keijsers RRMC Hendriks AGM van Erp PEJ van Cranenbroek B van de Kerkhof PCM Koenen HJPM In vivo induction of cutaneous inflammation results in the accumulation of extracellular trap-forming neutrophils expressing RORγt and IL-17. J Invest Dermatol (2014) 134(5):1276–84. 10.1038/jid.2013.526 24317395

[125] Leppkes M Maueröder C Hirth S Nowecki S Günther C Billmeier U Externalized decondensed neutrophil chromatin occludes pancreatic ducts and drives pancreatitis. Nat Commun (2016) 7:10973. 10.1038/ncomms10973 26964500 PMC4793047

[126] Martin WJ Walton M Harper J. Resident macrophages initiating and driving inflammation in a monosodium urate monohydrate crystal-induced murine peritoneal model of acute gout. Arthritis Rheum (2009) 60(1):281–9. 10.1002/art.24185 19116939

[127] Liu-Bryan R Scott P Sydlaske A Rose DM Terkeltaub R. Innate immunity conferred by Toll-like receptors 2 and 4 and myeloid differentiation factor 88 expression is pivotal to monosodium urate monohydrate crystal-induced inflammation. Arthritis Rheum (2005) 52(9):2936–46. 10.1002/art.21238 16142712

[128] Scott P Ma H Viriyakosol S Terkeltaub R Liu-Bryan R. Engagement of CD14 mediates the inflammatory potential of monosodium urate crystals. J Immunol (2006) 177(9):6370–8. 10.4049/jimmunol.177.9.6370 17056568

[129] Mitroulis I Kambas K Ritis K. Neutrophils, IL-1β, and gout: is there a link? Semin Immunopathol (2013) 35(4):501–12. 10.1007/s00281-013-0361-0 23344781

[130] Terkeltaub R. Update on gout: new therapeutic strategies and options. Nat Rev Rheumatol (2010) 6(1):30–8. 10.1038/nrrheum.2009.236 20046204

[131] Schorn C Janko C Krenn V Zhao Y Munoz LE Schett G Bonding the foe – NETting neutrophils immobilize the pro-inflammatory monosodium urate crystals. Front Immunol (2012) 3:376. 10.3389/fimmu.2012.00376 23233855 PMC3517988

[132] Maueröder C Kienhöfer D Hahn J Schauer C Manger B Schett G How neutrophil extracellular traps orchestrate the local immune response in gout. J Mol Med (Berl) (2015) 93(7):727–34. 10.1007/s00109-015-1295-x 26002146

[133] Mitroulis I Skendros P Ritis K. Targeting IL-1beta in disease; the expanding role of NLRP3 inflammasome. Eur J Intern Med (2010) 21(3):157–63. 10.1016/j.ejim.2010.03.005 20493414

[134] Dinarello CA van der Meer JWM. Treating inflammation by blocking interleukin-1 in humans. Semin Immunol (2013) 25(6):469–84. 10.1016/j.smim.2013.10.008 24275598 PMC3953875

[135] Kahn SE Cooper ME Del Prato S. Pathophysiology and treatment of type 2 diabetes: perspectives on the past, present, and future. Lancet (2014) 383(9922):1068–83. 10.1016/S0140-6736(13)62154-6 24315620 PMC4226760

[136] Joshi MB Lad A Bharath Prasad AS Balakrishnan A Ramachandra L Satyamoorthy K. High glucose modulates IL-6 mediated immune homeostasis through impeding neutrophil extracellular trap formation. FEBS Lett (2013) 587(14):2241–6. 10.1016/j.febslet.2013.05.053 23735697

[137] Wong SL Demers M Martinod K Gallant M Wang Y Goldfine AB Diabetes primes neutrophils to undergo NETosis, which impairs wound healing. Nat Med (2015) 21(7):815–9. 10.1038/nm.3887 26076037 PMC4631120

[138] Huang J Xiao Y Xu A Zhou Z. Neutrophils in type 1 diabetes. J Diabetes Investig (2016) 7(5):652–63. 10.1111/jdi.12469 27181374 PMC5009125

[139] Qin J Fu S Speake C Greenbaum CJ Odegard JM. NETosis-associated serum biomarkers are reduced in type 1 diabetes in association with neutrophil count. Clin Exp Immunol (2016) 184(3):318–22. 10.1111/cei.12783 26939803 PMC4872375

[140] Roche N Marthan R Berger P Chambellan A Chanez P Aguilaniu B Beyond corticosteroids: future prospects in the management of inflammation in COPD. Eur Respir Rev (2011) 20(121):175–82. 10.1183/09059180.00004211 21881145 PMC9584116

[141] Papayannopoulos V Staab D Zychlinsky A. Neutrophil elastase enhances sputum solubilization in cystic fibrosis patients receiving DNase therapy. PLoS One (2011) 6(12):e28526. 10.1371/journal.pone.0028526 22174830 PMC3235130

[142] Milla CE Recombinant human DNase in cystic fibrosis. Lancet (1999) 354(9176):428 10.1016/S0140-6736(05)75848-7 10437902

[143] Pressler T. Review of recombinant human deoxyribonuclease (rhDNase) in the management of patients with cystic fibrosis. Biologics (2008) 2(4):611–7. 10.2147/BTT.S3052 19707442 PMC2727891

[144] Caudrillier A Kessenbrock K Gilliss BM Nguyen JX Marques MB Monestier M Platelets induce neutrophil extracellular traps in transfusion-related acute lung injury. J Clin Invest (2012) 122(7):2661–71. 10.1172/JCI61303 22684106 PMC3386815

[145] Dubois AV Gauthier A Bréa D Varaigne F Diot P Gauthier F Influence of DNA on the activities and inhibition of neutrophil serine proteases in cystic fibrosis sputum. Am J Respir Cell Mol Biol (2012) 47(1):80–6. 10.1165/rcmb.2011-0380OC 22343221

[146] Berger-Achituv S Brinkmann V Abed UA Kühn LI Ben-Ezra J Elhasid R A proposed role for neutrophil extracellular traps in cancer immunoediting. Front Immunol (2013) 4:48. 10.3389/fimmu.2013.00048 23508552 PMC3589747

[147] Demers M Wagner DD. Neutrophil extracellular traps: a new link to cancer-associated thrombosis and potential implications for tumor progression. Oncoimmunology (2013) 2(2):e22946. 10.4161/onci.22946 23526174 PMC3601165

[148] Cools-Lartigue J Spicer J Najmeh S Ferri L. Neutrophil extracellular traps in cancer progression. Cell Mol Life Sci (2014) 71(21):4179–94. 10.1007/s00018-014-1683-3 25070012 PMC7096049

[149] Wculek SK Malanchi I. Neutrophils support lung colonization of metastasis-initiating breast cancer cells. Nature (2015) 528(7582):413–7. 10.1038/nature16140 26649828 PMC4700594

[150] Arelaki S Arampatzioglou A Kambas K Papagoras C Miltiades P Angelidou I Gradient infiltration of neutrophil extracellular traps in colon cancer and evidence for their involvement in tumour growth. PLoS One (2016) 11(5):e0154484. 10.1371/journal.pone.0154484 27136460 PMC4852909

[151] Ho-Tin-Noé B Carbo C Demers M Cifuni SM Goerge T Wagner DD. Innate immune cells induce hemorrhage in tumors during thrombocytopenia. Am J Pathol (2009) 175(4):1699–708. 10.2353/ajpath.2009.090460 19729481 PMC2751565

[152] Mishalian I Bayuh R Levy L Zolotarov L Michaeli J Fridlender ZG. Tumor-associated neutrophils (TAN) develop pro-tumorigenic properties during tumor progression. Cancer Immunol Immunother (2013) 62(11):1745–56. 10.1007/s00262-013-1476-9 24092389 PMC11028422

[153] Sangaletti S Tripodo C Vitali C Portararo P Guarnotta C Casalini P Defective stromal remodeling and neutrophil extracellular traps in lymphoid tissues favor the transition from autoimmunity to lymphoma. Cancer Discov (2014) 4(1):110–29. 10.1158/2159-8290.CD-13-0276 24189145

[154] Narasaraju T Yang E Samy RP Ng HH Poh WP Liew A-A Excessive neutrophils and neutrophil extracellular traps contribute to acute lung injury of influenza pneumonitis. Am J Pathol (2011) 179(1):199–210. 10.1016/j.ajpath.2011.03.013 21703402 PMC3123873

[155] McDonald B Urrutia R Yipp BG Jenne CN Kubes P. Intravascular neutrophil extracellular traps capture bacteria from the bloodstream during sepsis. Cell Host Microbe (2012) 12(3):324–33. 10.1016/j.chom.2012.06.011 22980329

[156] Meng W Paunel-Görgülü A Flohé S Hoffmann A Witte I MacKenzie C Depletion of neutrophil extracellular traps in vivo results in hypersusceptibility to polymicrobial sepsis in mice. Crit Care (2012) 16(4):R137. 10.1186/cc11442 22835277 PMC3580722

[157] Hsu RYC Chan CHF Spicer JD Rousseau MC Giannias B Rousseau S LPS-induced TLR4 signaling in human colorectal cancer cells increases beta1 integrin-mediated cell adhesion and liver metastasis. Cancer Res (2011) 71(5):1989–98. 10.1158/0008-5472.CAN-10-2833 21363926

[158] McDonald B Spicer J Giannais B Fallavollita L Brodt P Ferri LE. Systemic inflammation increases cancer cell adhesion to hepatic sinusoids by neutrophil mediated mechanisms. Int J Cancer (2009) 125(6):1298–305. 10.1002/ijc.24409 19431213

[159] Spicer JD McDonald B Cools-Lartigue JJ Chow SC Giannias B Kubes P Neutrophils promote liver metastasis via Mac-1-mediated interactions with circulating tumor cells. Cancer Res (2012) 72(16):3919–27. 10.1158/0008-5472.CAN-11-2393 22751466

[160] Liang S Hoskins M Khanna P Kunz RF Dong C. Effects of the tumor-leukocyte microenvironment on melanoma-neutrophil adhesion to the endothelium in a shear flow. Cell Mol Bioeng (2008) 1(2–3):189–200. 10.1007/s12195-008-0016-8 19865613 PMC2768417

[161] Barnado A Crofford LJ Oates JC. At the bedside: neutrophil extracellular traps (NETs) as targets for biomarkers and therapies in autoimmune diseases. J Leukoc Biol (2016) 99(2):265–78. 10.1189/jlb.5BT0615-234R 26658004 PMC6608010

[162] Costedoat-Chalumeau N Dunogué B Morel N Le Guern V Guettrot-Imbert G. Hydroxychloroquine: a multifaceted treatment in lupus. Presse Med (2014) 43(6 Pt 2):e167–80. 10.1016/j.lpm.2014.03.007 24855048

[163] Garcia RJ Francis L Dawood M Lai Z Faraone SV Perl A. Attention deficit and hyperactivity disorder scores are elevated and respond to N-acetylcysteine treatment in patients with systemic lupus erythematosus. Arthritis Rheum (2013) 65(5):1313–8. 10.1002/art.37893 23400548 PMC4034122

[164] Khamashta M Merrill JT Werth VP Furie R Kalunian K Illei GG Sifalimumab, an anti-interferon-α monoclonal antibody, in moderate to severe systemic lupus erythematosus: a randomised, double-blind, placebo-controlled study. Ann Rheum Dis (2016) 75(11):1909–16. 10.1136/annrheumdis-2015-208562 27009916 PMC5099191

[165] Knight JS Zhao W Luo W Subramanian V O’Dell AA Yalavarthi S Peptidylarginine deiminase inhibition is immunomodulatory and vasculoprotective in murine lupus. J Clin Invest (2013) 123(7):2981–93. 10.1172/JCI67390 23722903 PMC3696545

[166] Willis VC Gizinski AM Banda NK Causey CP Knuckley B Cordova KN N-α-benzoyl-N5-(2-chloro-1-iminoethyl)-l-ornithine amide, a protein arginine deiminase inhibitor, reduces the severity of murine collagen-induced arthritis. J Immunol (2011) 186(7):4396–404. 10.4049/jimmunol.1001620 21346230 PMC3085980

[167] Lewis HD Liddle J Coote JE Atkinson SJ Barker MD Bax BD Inhibition of PAD4 activity is sufficient to disrupt mouse and human NET formation. Nat Chem Biol (2015) 11(3):189–91. 10.1038/nchembio.1735 25622091 PMC4397581

[168] Patel T Gordon KB. Adalimumab: efficacy and safety in psoriasis and rheumatoid arthritis. Dermatol Ther (2004) 17(5):427–31. 10.1111/j.1396-0296.2004.04045.x 15379777

[169] Totani L Amore C Di Santo A Dell’Elba G Piccoli A Martelli N Roflumilast inhibits leukocyte-platelet interactions and prevents the prothrombotic functions of polymorphonuclear leukocytes and monocytes. J Thromb Haemost (2016) 14(1):191–204. 10.1111/jth.13173 26484898

[170] Zeerleder S van Bijnen S Wouters D van Mierlo GJ Muus P Neutrophil extracellular trap formation in PNH patients with and without a history of thrombosis – effects of eculizumab. Blood (2013) 122(21):1235.

[171] Gavillet M Martinod K Renella R Harris C Shapiro NI Wagner DD Flow cytometric assay for direct quantification of neutrophil extracellular traps in blood samples. Am J Hematol (2015) 90(12):1155–8. 10.1002/ajh.24185 26347989 PMC4715743

